# Scalable excitatory synaptic circuit design using floating gate based leaky integrators

**DOI:** 10.1038/s41598-017-17889-8

**Published:** 2017-12-14

**Authors:** Vladimir Kornijcuk, Hyungkwang Lim, Inho Kim, Jong-Keuk Park, Wook-Seong Lee, Jung-Hae Choi, Byung Joon Choi, Doo Seok Jeong

**Affiliations:** 1 Center for Electronic Materials, Korea Institute of Science and Technology, Seoul, 02792 Republic of Korea; 2 Department of Nanomaterials, University of Science and Technology, Daejeon, 34113 Republic of Korea; 3 Department of Materials Science and Engineering, Seoul National University of Science and Technology, Seoul, 01811 Republic of Korea

## Abstract

We propose a scalable synaptic circuit realizing spike timing dependent plasticity (STDP)—compatible with randomly spiking neurons. The feasible working of the circuit was examined by circuit simulation using the BSIM 4.6.0 model. A distinguishable feature of the circuit is the use of floating-gate integrators that provide the compact implementation of biologically plausible relaxation time scale. This relaxation occurs on the basis of charge tunneling that mainly relies upon area-independent tunnel barrier properties (e.g. barrier width and height) rather than capacitance. The circuit simulations feature (i) weight-dependent STDP that spontaneously limits the synaptic weight growth, (ii) competitive synaptic adaptation within both unsupervised and supervised frameworks with randomly spiking neurons. The estimated power consumption is merely 34 pW, perhaps meeting one of the most crucial principles (power-efficiency) of neuromorphic engineering. Finally, a means of fine-tuning the STDP behavior is provided.

## Introduction

For nearly three decades, the brain and its information processing principles have been a benchmark in building artificial intelligence (AI) that enables recognition tasks by means of very-large-scale integration (VLSI) technology—often referred to as neuromorphic engineering^1^. Attention to this has recently been boosted with regard to remarkably growing demands for hardware-based AI systems. The early attempts mostly revolved around realizing scalable replicas of biological spiking units (neurons)^2,3^ and their application to front-end sensors, e.g. silicon retinas^4^. These early attempts were then followed by a number of spiking neuron models with different degrees of biological plausibility, complexity, and tunability^5–11^, enriching available neuron models. Essential to neuromorphic engineering for AI are memory and learning that are believed to involve synaptic weight modification in support of feature abstraction. Spike timing dependent plasticity (STDP) is a seminal learning rule that describes the causality of postsynaptic spiking in a time domain^12–15^. Frequently, neuromorphic engineers benchmark the STDP in view of, mostly, its capability of temporal learning (real-time learning) and compatibility with neuromorphic systems^16–20^.

The STDP relates the long-lasting change of synaptic weight *w* to the temporal order between pre- and postsynaptic spike times (Δ*t* = *t*_post_ − *t*_pre_); long-term potentiation (LTP) is induced when the presynaptic spike precedes the postsynaptic one, and long-term depression (LTD) in the opposite case. For convenience, the former configuration of spikes is denoted by pre-post, and the latter post-pre. Mathematically, the STDP is often simplified as

1 $${\rm{\Delta }}w({\rm{\Delta }}t)=\{\begin{array}{ll}{A}_{+}{s}_{{\rm{pre}}}({\rm{\Delta }}t) & {\rm{if}}\,{\rm{\Delta }}t > 0\\ -{A}_{-}{s}_{{\rm{post}}}(-{\rm{\Delta }}t) & {\rm{if}}\,{\rm{\Delta }}t < 0\\ 0 & {\rm{if}}\,{\rm{\Delta }}t=0,\end{array}$$

where *s*_pre_ and *s*_post_ are pre- and postsynaptic state variables that exponentially decay with Δ*t* at likely different time constants (*τ*_+_ and *τ*_−_), defining the degree of LTP and LTD, respectively. *A*_+_ and *A*_−_ define the maximum weight change. They can be either constant or reliant upon the current weight, which causes a significant difference in synaptic weight evolution through a learning period^21–24^.

A typical strategy for realizing the STDP in a neuromorphic circuit is to deploy two leaky integrators for pre and postsynaptic state variables (*s*_pre_ and *s*_post_) in conjunction with an analog or digital memory unit to store the evaluated synaptic weight. This general framework has been applied to various synaptic circuit designs thus far; a good review is given by Bamford *et al*.^17^. Table 1 summarizes several previous STDP circuits that are capable of real-time scale operation. Notably, the state variables are often realized by (i) leaky voltage integrators using a standalone capacitor or the gate capacitor of a transistor (switched-capacitor integrators)^17,25,26^, (ii) current-starved inverter^27^, and (iii) operational transconductance amplifier (OTA)-based integrator^19,28^. Scaling down a metal-oxide-semiconductor field-effect transistor (MOSFET) in the integrator (particularly channel length below 100 nm) causes a significant rise in subthreshold leakage current^29–31^, and thus a large decrease in the relaxation time of the integrator with a given capacitor. For temporal learning, the relaxation time is *a priori* preferred to be comparable to that of the biological counterpart in favor of energy-efficient learning, sacrificing unnecessarily fast response. In this regard, needs for higher capacitance to compensate for the subthreshold leakage—maintaining the biologically plausible relaxation time—perhaps limit further scaling down. A workaround is to adopt digital technologies as done by Vogelstein *et al*.^32^; a random access memory (RAM) was deployed to store discrete state variable values, and their updates were evaluated in a programmable manner using a microcontroller unit (MCU). Upon every spiking event, the MCU scans the entire RAM and updates the synaptic weights according to the STDP rule. As such, this digital implementation readily offers flexibility in designing the STDP model, hence can serve as a convenient platform in combination with hardware neurons. A possible disadvantage is, however, such that the weight values in the RAM are updated in serial order (time consuming), which hinders a large network with a number of connections (synapses) from real-time interaction with physical environments.

**Table 1:** Summary of previous STDP circuit designs.

Reference	Weight	State variable implementation	Weight storage element	Technology
S. A. Bamford *et al*.^17^	Analog	Capacitor-based leaky integrator	Capacitor	0.35 um
G. Indiveri *et al*.^26^	Analog-bistable	Capacitor-based leaky integrator	Capacitor	0.80 um
J. V. Arthur and K. Boahen (2006)^25^	Binary	Capacitor-based leaky integrator	SRAM	0.25 um
S. Ramakrishnan *et al*.^27^	Analog	Current-starved inverter	FG transistor	0.35 um
A. Bofill-i-Petit *et al*.^19^	Analog	OTA-based leaky integrator	Capacitor	0.60 um
J. M. Cruz-Albrecht^28^	Analog	OTA-based leaky integrator	Capacitor	90 nm
R. J. Vogelstein *et al*. (2002)^32^	Quasi-analog (discretized)	Programmed into the microcontroller	RAM	—
This work	Analog	FG-based leaky integrator	FG transistor	65 nm (simulation)

Another important aspect of synaptic circuit design is synaptic weight storage. Ideally, each synaptic unit has a long-lasting analog weight value in a desired range. A common strategy is to use a standalone capacitor that enables current integration and consequently outputs analog voltage^17,19,28^. However, it is challenging to achieve ideal weight storage because of information loss in due course. The charge loss (poor retention) given the leakage in the subthreshold MOSFET is generally a downside of this common strategy. As a workaround, long-term storage is offered by a bistability circuit that drives the capacitor voltage to one of two stable states^26^ or by a RAM that stores binary weight values^25^. Regarding the latter approach, the stored weight value is not necessarily binary. An analog-digital converter can be used to digitize the value that is subsequently stored in a RAM as mentioned above^32^, though the memory capacity restricts the precision of weight. Floating-gate (FG) based synaptic circuits may meet the requirements, which offer both long-lasting storage and analog-type weight representation^33^. A gate voltage in a floating-gate MOSFET (FG-MOSFET) is in control of the charge on the FG—the charge can be maintained in the standby state—which alters the channel conductance. Following the original proposal, the design was refined by Ramakrishnan *et al*.^27^ and Brink *et al*.^34^, offering a viable solution to VLSI synapse design. Other than these mainstream strategies, an emerging approach offers the feasible use of resistive RAM (RRAM for short or popularly referred to as memristive device) based on novel materials as a memory bit^35–41^. RRAM exhibits nonvolatile resistive switching between multinary states (not all types of RRAMs though). Additionally, an RRAM array (particularly, passive crossbar array) is highly scalable, meeting the design rule of 4*F* ^2^ for the passive array.

In this work, we propose a VLSI-compatible synaptic circuit for spiking neural network, which captures the pair-based STDP behavior^13^. This synaptic circuit was designed by adopting 65 nm CMOS technology and its feasible operation was examined by using the BSIM 4.6.0 model^42^ with foundry parameters—a built-in model in LTspice IV. The circuit employs three FG-MOSFETs whose function is two-fold: two FG-based leaky integrators to realize pre and postsynaptic state variables (*s*_pre_ and *s*_post_) and an additional FG-MOSFET to store the weight value. The first two FG-MOSFETs differ in retention time from the last one; the charge on the FG in each of them is released at a biologically plausible rate, whereas the last one needs to be of long retention. For this storage FG-MOSFET, the detailed balance between charge injection into and ejection out of the FG limits the growth of synaptic weight, leaving the coefficients *A* ^+^ and *A* ^−^ in (1) dependent upon synaptic weight. Eventually, we pay attention to the competitive adaptation of synaptic weight within unsupervised and supervised frameworks and the detailed kinetics of the adaptation by phase-plane analysis. The proposed synaptic circuit appears to host such synaptic functions.

## Results

### FG synaptic circuit

**Figure 1:**
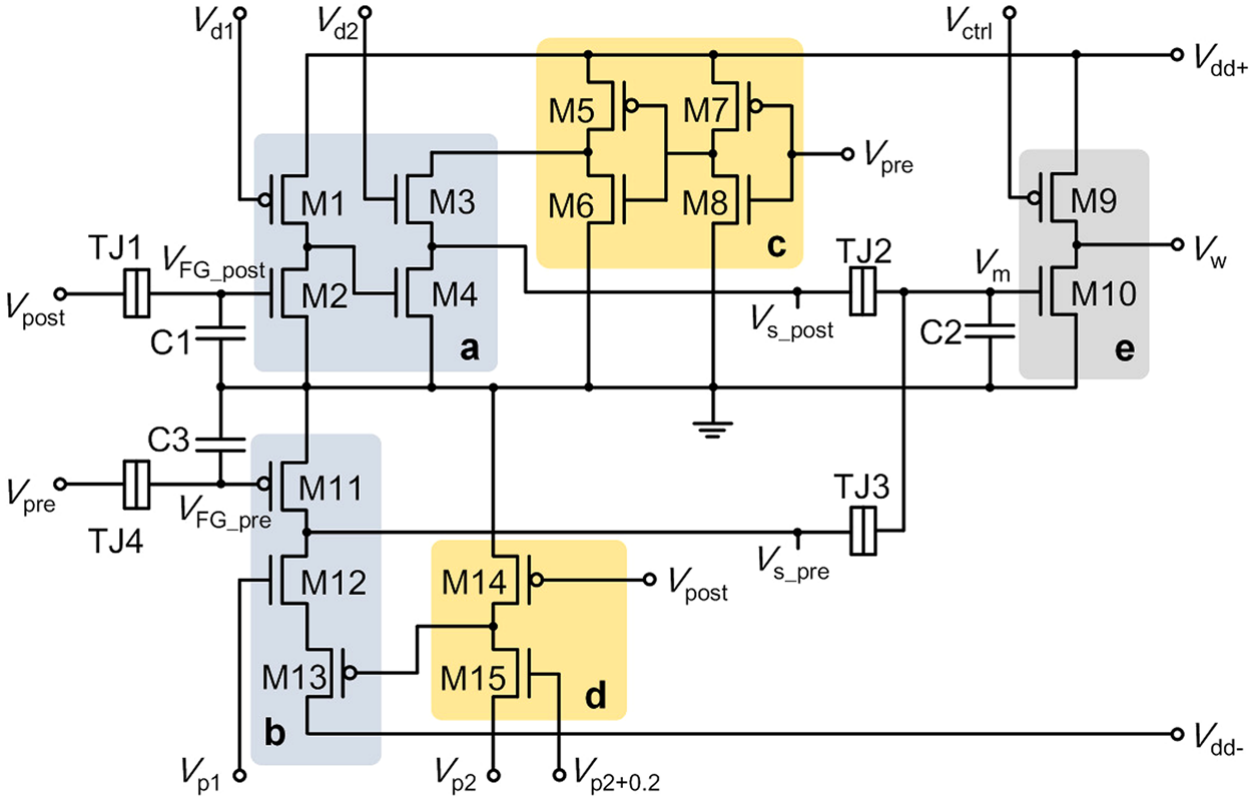
Proposed synaptic circuit. Leaky integration is realized on the FG of M2 and M11, which is incorporated into the state variable generators (**a**) and (**b**) for post and presynaptic variable, respectively. Sampling subcircuits (**c**) and (**d**) read out the current post and presynaptic variable, respectively, and relay them to (**e**) the storage subcircuit. This storage subcircuit converts *V*_m_ to synaptic weight that is parameterized by *V*_w_.

The FG synaptic circuit is shown in Fig. 1. This circuit realizes the pre and postsynaptic state variables (*s*_pre_ and *s*_post_), and they are determined by spiking history. Note that considered is nearest-spike interaction between pre and postsynaptic spikes^43^. Evaluating the state variables follows the two steps: (i) introducing a continuously varying function that outputs each state variable and (ii) sampling the value upon an incoming spike. The pre and postsynaptic state variables are parameterized by *V*_s_pre_ and *V*_s_post_, respectively. A positive weight change in LTP is dictated by the presynaptic state variable while a negative change in LTD by postsynaptic state variable, and thereby it is intuitive to endow *V*_s_pre_ and *V*_s_post_ with different polarities (here *V*_s_pre_ ≤ 0 and *V*_s_post_ ≥ 0). For the postsynaptic state variable, the subcircuit in Fig. 1a (leaky integrator) takes up the first task (introduction of a state variable function), and that in Fig. 1c (sampling) samples the current value only if a presynaptic spike is applied. For the presynaptic state variable, the subcircuits in Fig. 1b and d introduce a state variable function and sample the current value, respectively.

Each leaky integrator comprises a FG-MOSFET (TJ1 + M2 and TJ4 + M11 for the post and presynaptic state variable, respectively) and voltage divider. Notably, for the postsynaptic state variable, the integrator has two stages for non-inverting voltage transfer characteristic (VTC). Each FG-MOSFET is a tunnel junction (TJ)-MOSFET stack in conjunction with an auxiliary capacitor (C1 and C3 for the post and presynaptic state variable, respectively) that is used for precisely initiating the desired charge relaxation. Each integrator piles up charge on the FG upon incident spikes in balance with charge relaxation (ejection) so that the channel conductance of the FG-MOSFET varies accordingly.

The sampling subcircuit for each variable outputs nonzero voltage only in the presence of a counter spike, i.e. sampling *s*_pre_ and *s*_post_ needs a post and a presynaptic spike, respectively. The nonzero output from the subcircuit reads the current state variable (*V*_s_pre_ and *V*_s_post_) of different polarities and relays it to the weight storage subcircuit.

The synaptic weight is memorized on the FG of the FG-MOSFET (TJ2 + TJ3 + M10). *V*_s_pre_ and *V*_s_post_ are applied to TJ3 and TJ2, respectively, and charge is accordingly integrated on the FG, outputting *V*_w_ in combination with the voltage divider M9. The auxiliary capacitor C2 is used as for the integrators. *V*_w_ is subsequently sampled by a presynaptic spike and applied to the membrane of the postsynaptic neuron, raising the membrane potential.

Note that the circuit parameters in Tables 2 and 3 were used for the simulations unless otherwise stated. The subcircuit-wise synaptic circuit operation is fully detailed in Supplementary Information.

**Table 2:** Parameters used for circuit simulations.

Spike amplitude (V)	Spike width (μs)	*C*_1_ (fF)	*C*_2_ (fF)	*C*_3_ (fF)	*V*_d1_ (V)	*V*_d2_ (V)
0.5	30	25	30	1.5	0.70	0.65
***V***_**p1**_ **(V)**	***V***_**p2**_ **(V)**	***V***_**ctrl**_ **(V)**	***V***_**dd+**_ **(V)**	***V***_**dd−**_ **(V)**	**Temperature**	
−0.26	−0.80	0.51	0.5	−0.5	27 °C

**Table 3:** Sizes of MOSFETs in use.

Subcircuit	Element number	Channel length (nm)	Channel width (nm)	Oxide thickness (nm)
FG nodes	TJ1, TJ4	60	120	1.1
	TJ2	60	120	1.75
	TJ3	120	240	1.75
Postsynaptic state variable generator	M1-M4	60	120	2.5
Postsynaptic state variable sampling	M5-M6	120	120	
	M7-M8	60	120	
Presynaptic state variable generator	M11-M12	60	120	
	M11	240	120	
Presynaptic state variable sampling	M14-M15	60	120	
Storage	M9	120	480	
	M10	90	1400	

### Plasticity induction

**Figure 2:**
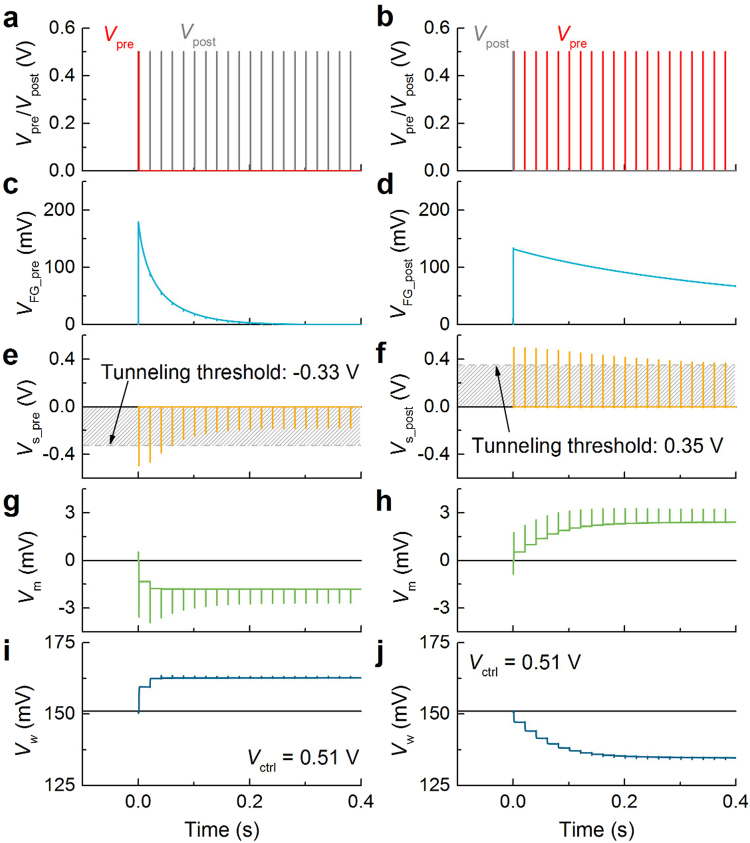
Simulated transient voltage characteristics during plasticity induction for (**a**) causal and (**b**) anti-causal spike patterns. The transient FG voltage following the first spike for both causal and anti-causal cases is plotted in (**c**) and (**d**), respectively. Given the spike pattern, the state variable is continuously elicited as shown in (**e**) and (**f**) and consequently alters the storage FG voltage (*V*_m_) [(**g**) and (**h**)], respectively. Eventually, synaptic weight *V*_w_ evolves as plotted in (**i**) and (**j**), indicating potentiation and depression, respectively.

The STDP behavior of the proposed circuit was first identified in a time domain for two preliminary cases (causal and anti-causal cases) using the circuit parameters listed in Tables 2 and 3. The former indicates a synapse subject to a single presynaptic spike that precedes a postsynaptic spike train (pre-post), whereas the latter the opposite order (post-pre) as plotted in Fig. 2a and b, respectively. The first pre and postsynaptic spikes abruptly raise the corresponding FG voltage by approximately 180 and 135 mV, respectively (Fig. 2c and d). The amplitude and width of each spike were 0.5 V and 30 μs, respectively. Note that *V*_FG_post_ has the larger relaxation time than *V*_FG_pre_, which endows the STDP behavior in a timing-difference (*t*_post_ − *t*_pre_) domain with the larger depression window than potentiation window as will be discussed below. *V*_FG_pre_ and *V*_FG_post_ were subsequently sampled by the following opposite spike trains, resulting in the state variables *V*_s_pre_ and *V*_s_post_, respectively (Fig. 2e and f). The sampled values were respectively applied to the tunneling junctions TJ2 and TJ3, causing the increase of *V*_m_ as shown in Fig. 2g and h. Notably, *V*_s_pre_ and *V*_s_post_ larger than a certain threshold contribute to the *V*_m_ change (Fig. 2g and h). This threshold is defined as voltage enabling the injection of one elementary charge for 30 μs—no noticeable change in *V*_m_ results from voltage below this threshold. Given the difference in size for TJ2 and TJ3 (Table 3), the threshold voltage also differs: 0.35 V and −0.33 V for TJ2 and TJ3, respectively. The synaptic weight *V*_w_ eventually varied upon the *V*_m_, which captures the synaptic plasticity upon the temporal order of pre and postsynaptic spikes (Fig. 2i and j). It is noted that the causal spike order (Fig. 2a) results in an increase in *V*_w_, i.e. potentiation while the anti-causal order in a decrease in *V*_w_, i.e. depression.

**Figure 3:**
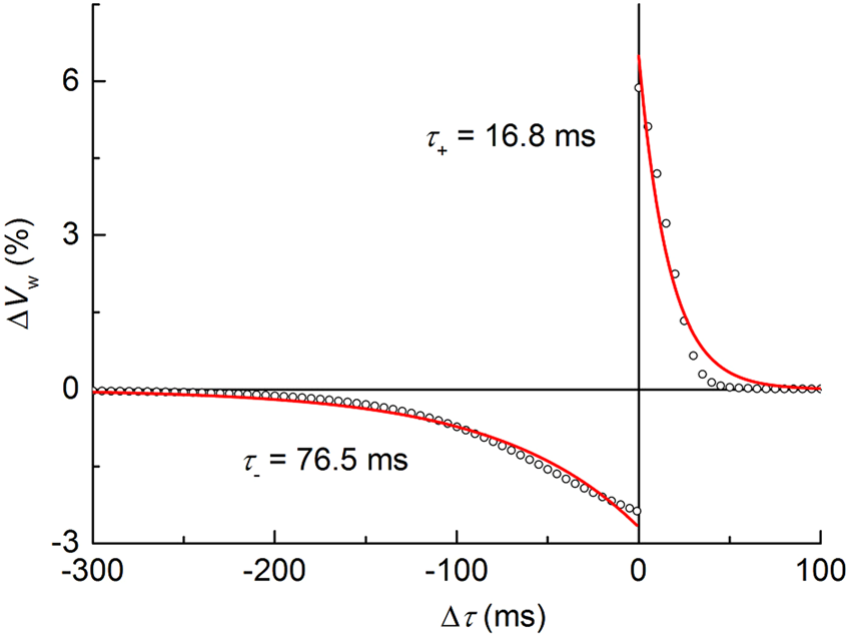
STDP behavior in a timing-difference domain. The change in synaptic weight (Δ*V*_w_) was evaluated with a single pair of pre and postsynaptic spikes (different Δ*t*). The circles indicate the simulation data, which are fitted to the simplified mathematical formula in (1) (solid red lines). The fitting results in *τ*_+_ and *τ*_−_ of 16.8 and 76.5 ms, respectively. The reference (initial) synaptic weight (*V*_w0_) was 151 mV that results from a *V*_ctrl_ of 0.51 V.

The proposed synaptic circuit with the same circuit parameters causes a STDP behavior in a timing-difference domain as plotted in Fig. 3. The reference (initial) weight (*V*_w0_) was approximately 151 mV. The LTD window is wider than the LTP; fitting the plot to (1) relates a LTD and LTP time constant of approximately 76.5 and 16.8 ms, respectively. The STDP behavior can be tweaked by means of circuit parameters as detailed in Supplementary Information.

### Weights dependence of STDP

It is important to delimit synaptic weight such that the uncontrolled growth is avoided. To do so, the weight change for each pair of pre and postsynaptic spikes needs to depend upon the current weight in a way that the increase declines with the increase of the current weight. In fact, the weight dependence of STDP has been demonstrated to be of importance in its functionalities on a network scale, such as selectivity development^21^, temporal correlation encoding^22^, receptive field stability^23^, and synaptic weight distributions^24^. The proposed circuit spontaneously leads to weight-depending STDP given (i) the detailed balance governing the charge transfer into and out of the FG in the storage element (TJ2 + TJ3 + M10) and (ii) output saturation in the VTC of M9 + M10 (see Supplementary Information). Given the detailed balance of charge transfer, the more electrons the FG keeps, the more negatively the FG (*V*_m_) is charged and thus the more likely the potential configuration repels further electron injection (see Supplementary Information). Consequently, the lower *V*_m_ for the moment, the more likely that a decrease in *V*_m_ by the next pre-post spike pair tends to be small, relating Δ*V*_m_ to the current synaptic weight. Besides, the common node (*V*_m_) for both LTP and LTD couples the weight-depending LTP and LTD such that Δ*V*_m_ for LTD (i.e. Δ*V*_m_ > 0) also depends upon the current *V*_m_. The lower *V*_m_ (<0) for the moment, the larger Δ*V*_m_ (Δ*V*_m_ > 0) tends to be caused by a post-pre spike pair. Alongside the detailed balance of charge dynamics, the VTC of the storage subcircuit—outputting voltage in 0–0.5 V as addressed in Supplementary Information—underpins the weight-depending LTP, particularly, limited growth of synaptic weight below a *V*_w_ of 0.5 V. Additionally, the VTC restricts *V*_w_ (≥0) such that the LTD that outweighs the LTP cannot lead to negative output (*V*_w_ < 0), *V*_w_ = 0 instead—implying no synaptic transmission.

**Figure 4:**
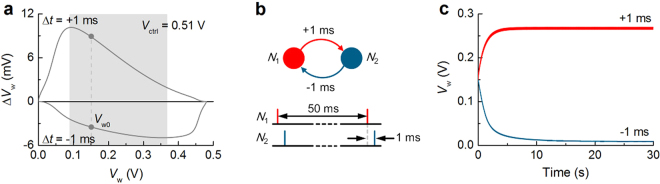
Simulated weight-depending STDP behavior of the proposed synaptic circuit. (**a**) Dependence of Δ*V*_w_ on *V*_w_ when the synaptic circuit is subject to a single pair of pre and postsynaptic spikes with Δ*t* = ±1 ms. In the gray region, Δ*V*_*w*_ is mostly governed by the detailed balance of charge transfer via the FG in the storage element, whereas out of the gray region the VTC of M9 + M10 mainly determines the weight dependence. (**b**) Schematic of bidirectionally wired neurons (*N*_1_ and *N*_2_) that fire correlated spikes with 1 ms Δ*t* every 50 ms. (**c**) Consequent synaptic bifurcation.

In support of the weight-depending STDP, Δ*V*_w_ when Δ*t* = ±1 ms was evaluated with *V*_w_ (Fig. 4). As such, Δ*V*_w_ significantly depends upon *V*_w_ due to the aforementioned two factors that simultaneously (but relatively) contribute to the weight-dependence. The gray region in Fig. 4a indicates the weight change that is dominantly impeded by the detailed balance insomuch as the VTC in the given *V*_w_ region is far from both output saturation regions (see Supplementary Information). In contrast, the VTC output saturation outweighs the detailed balance outside the gray region, resulting in Δ*V*_w_ falling to zero in the vicinity of the two poles (0 and 0.5 V).

This weight-depending STDP was applied to a preliminary system in which two neurons (*N*_1_ and *N*_2_) were bidirectionally coupled through the proposed synaptic circuit (Fig. 4b). A pair of spikes (*N*_1_’s spike preceding *N*_2_’s by 1 ms) was elicited every 50 ms (20 Hz). The two synapses accordingly adjusted their weight in the given circumstances as shown in Fig. 4c. It is clearly noticed that the persistent stimulation bifurcates the two synapses from the initial weight (ca. 151 mV) with regard to the temporal order of spikes; the synapse from *N*_1_ to *N*_2_ encountered spike pairs that support LTP (Δ*t* = 1 ms) while the other underwent LTD given that Δ*t* = −1 ms. Additionally, the synaptic weight saturation obviously reflects the weight-depending STDP. As shown in Fig. 4a, a pair of spikes (Δ*t* = 1 ms) causes a noticeable rise in *V*_w_ unless the current *V*_w_ is below approximately 0.45 V. By all rights, this value (0.45 V) is supposed to be the maximum *V*_w_. However, the actual maximum *V*_w_ is around 0.27 V. This inconsistency arises from the interaction between the postsynaptic spike in a pair and presynaptic spike in the next pair, which meets the LTD condition (Δ*t* = −49 ms). Given the wide time window for LTD (Fig. 3), Δt of −49 ms is sufficient for a notable decrease in *V*_w_. In this regard, the maximum *V*_w_ is determined mainly by the weight dependence of LTP, but in combination with LTD caused by the wide LTD time window.

The weight-dependence can be tweaked by means of control signal *V*_ctrl_, e.g. initial synaptic weight (*V*_w0_) and upper and lower limits of weight, and thus the desired STDP behavior can readily be achieved. We set aside this issue until Supplementary Information.

### Competition between synapses

**Figure 5:**
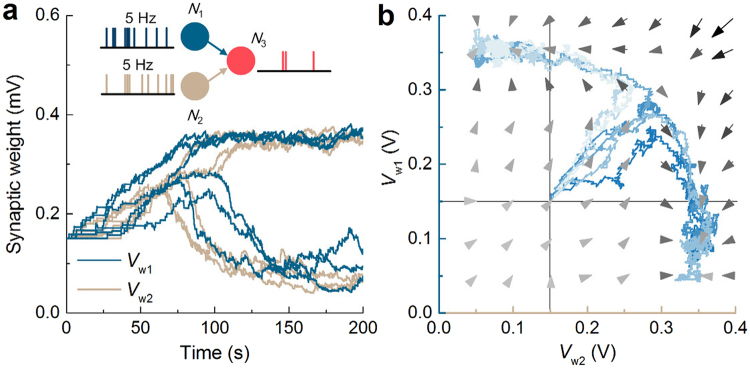
Unsupervised competition between two presynaptic neurons. (**a**) Synaptic evolution (adaptation) under temporal spiking configuration of two Poisson neurons (*N*_1_ and *N*_2_) and single postsynaptic neuron (*N*_3_) is plotted. The schematic of the network is depicted in the inset. *N*_1_ and *N*_2_ fire Poisson spikes at 5 Hz, and *N*_3_ spikes accordingly. (**b**) Phase plane analysis on synaptic bifurcation on the trials in (**a**)—indicated by different colors. The winning synapse was chosen at random. The same parameters as tabulated in Tables 2 and 3 were used.

The feasibility of the proposed synaptic circuit was further validated for a small network within the unsupervised and supervised learning frameworks. The test network was composed of two presynaptic (*N*_1_ and *N*_2_) and a single postsynaptic neuron (*N*_3_) as shown in the inset of Fig. 5a. The presynaptic neurons were Poisson neurons that spike following a renewal process (Poisson process). The procedure for Poisson spike generation can be seen in ref.^44^. The postsynaptic neuron was assumed to be a point neuron and realized by employing the Stein model^45^ in which the following subthreshold membrane potential *u*_m_ holds:

2 $${\tau }_{m}\frac{{\rm{d}}{u}_{{\rm{m}}}}{{\rm{d}}t}=-{u}_{{\rm{m}}}+\alpha \sum _{i=1}^{{N}_{u}}\sum _{j=0}^{{N}_{s}}{V}_{{\rm{w}}}^{i}(t)\delta (t-{t}_{j}^{i}),$$

where *τ*_*m*_ denotes the relaxation time constant of the membrane and was set to 10 ms. *N*_*u*_ and *N*_*s*_ mean the number of presynaptic neurons (here two) and the total number of spikes from each presynaptic neuron. The superscript indicates presynaptic neuron label such that ${V}_{{\rm{w}}}^{i}(t)$ and ${t}_{j}^{i}$ mean the synaptic weight for the presynaptic neuron (*i* = 1 or 2) and *j* ^th^ spiking time for the same presynaptic neuron, respectively. Thus, the Dirac delta function samples the synaptic weight in response to spiking, and the sampled value—multiplied by constant *α* (set to 0.3)—is a rise in *u*_m_ upon spiking. Once the threshold for spiking (90 mV) is reached, the neuron fires a spike and subsequently resets *u*_m_ to zero. The parameters in Tables 2 and 3 were used for these simulations.

First, we considered the synaptic weight evolution in response to uncorrelated Poisson spikes from *N*_1_ and *N*_2_ and the induced postsynaptic spikes from *N*_3_ (unsupervised learning). *N*_1_ and *N*_2_ were assumed to spike at 5 Hz. *N*_3_ receives the presynaptic spikes via the synaptic circuits, and the membrane potential consequently evolves. The change of *V*_w1_ (for *N*_1_ → *N*_3_) and *V*_w2_ (for *N*_2_ → *N*_3_) in these circumstances is displayed in Fig. 5a. The evolution can be divided into two phases, a simultaneous increase in weight in the first place (ca. 0–75 s) and the subsequent synaptic bifurcation. In the first phase, *N*_1_ and *N*_2_ together elicit spikes from *N*_3_, and their contribution is likely equal given the same spiking rate. That is, synaptic association is dominant over competition such that both synapses are reinforced given the causal order of pre-postsynaptic spikes for both synapses. Through this phase, each synaptic weight becomes sufficiently high to evoke a postsynaptic spike without association, which is then followed by competition, implying transition to the bifurcation phase (*t* > 75 s). The competition takes place at random as follows; (i) in view of the high synaptic weight one of *N*_1_ and *N*_2_ is solely able to evoke a postsynaptic spike, (ii) the causal correlation between either *N*_1_ or *N*_2_ (chosen at random) and *N*_3_ is consequently established, which reinforces the chosen synapse, and (iii) in contrast, the unchosen synapse is subject to uncorrelated pre and postsynaptic spiking. The uncorrelated spiking probabilistically makes the anti-causal effect dominant over the other in light of the larger time windows for LTD than LTP (see Fig. 3). Thus, the synapse falls behind in the competition.

The weight evolution from the initial value is better visualized on the *V*_w1_ − *V*_w2_ phase plane in Fig. 5b. This phase plane analysis helps us readily predict the dynamics of *V*_w1_ and *V*_w2_ changes and important states such as null-clines and fixed points (if exist). Each arrow on the plane denotes a vector field $(d{V}_{{\rm{w}}1}/dt)\overrightarrow{i}+(d{V}_{{\rm{w}}2}/dt)\overrightarrow{j}$ at a given (*V*_w1_, *V*_w2_) point. The vector indicates Δ*V*_w1_/Δ*t* and Δ*V*_w2_/Δ*t* from the current states (*V*_w1_(*t*), *V*_w2_(*t*)): Δ*V*_w1_/Δ*t* = [*V*_w1_(*t* + Δ*t*) − *V*_w1_(*t*)]/Δ*t* and Δ*V*_w2_/ΔΔΔ*t* = [*V*_w2_(*t* + Δ*t*) − *V*_w2_(*t*)]/Δ*t*. *V*_w1_(*t* + Δ*t*) and *V*_w2_(*t* + Δ*t*) were statistically evaluated with (*V*_w1_(*t*), *V*_w2_(*t*)) that were subject to Poisson presynaptic spikes at 5 Hz for 2 s (Δ*t* = 2). This field evaluated was repeated over all nodes on the plane, resulting in the phase plane. The datasets in Fig. 5a are re-plotted on the phase plane, where their evolutions are in good agreement with the vector fields. Notably, the phase plane is symmetric with respect to a diagonal given that *N*_1_ and *N*_2_ spike at the same rate (5 Hz). Thus, the opposite trajectories were observed at random with equal probability.

**Figure 6:**
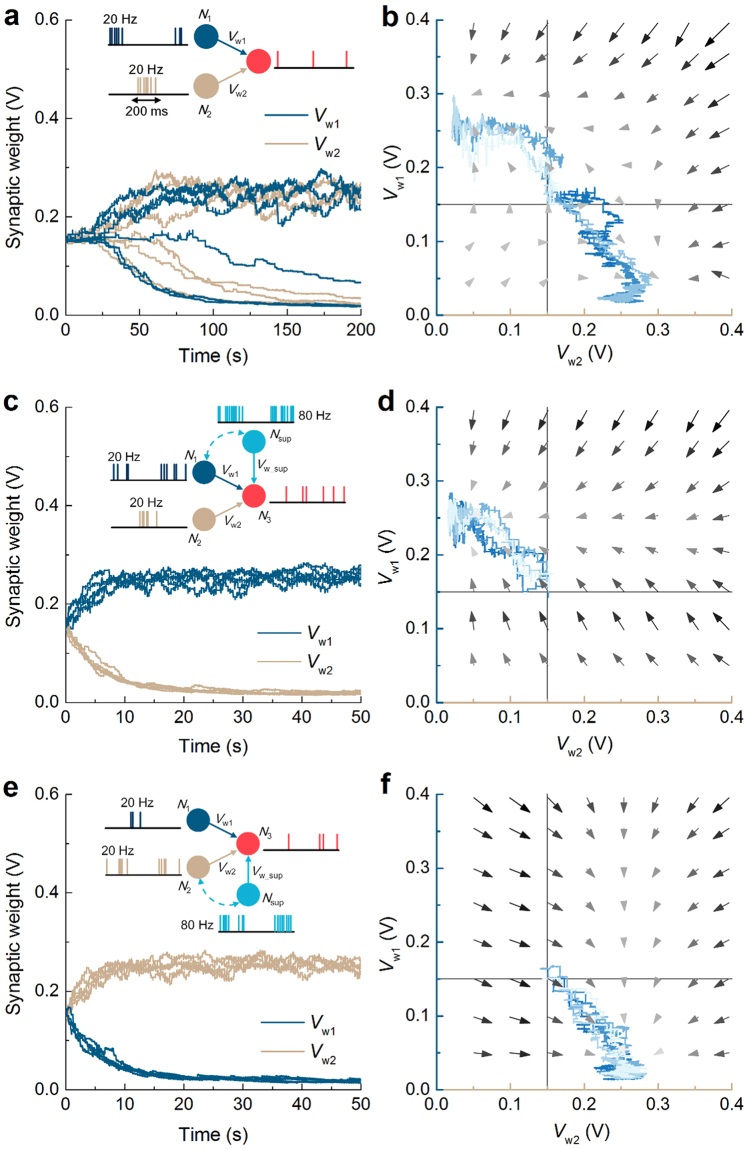
Comparison between unsupervised and supervised adaptation cases. (**a**) Unsupervised competition in the same network as the inset in Fig. 5(a). One of *N*_1_ and *N*_2_ was randomly chosen every time bin (width: 200 ms), and the chosen neuron was given a spiking rate of 20 Hz. Sampled synaptic evolution trajectories are plotted on the phase plane in (**b**)—each color denotes each sample. The two opposite types of trajectories were observed at random at the equal probabilities. (**c**) Supervised adaptation by deploying a bias neuron (*N*_sup_) that spikes at 80 Hz in sync with *N*_1_. With the aid of *N*_sup_, *N*_1_ always wins *N*_2_ as seen on the phase plane in (**d**). For *N*_sup_ in sync with *N*_2_, which makes *N*_2_ win *N*_1_, data are shown in (**e**) and (**f**).

Following is synaptic weight evolution upon time-varying spiking rate for the same simple network. In this simulation, only one of *N*_1_ and *N*_2_ fired spikes within a time bin of 200 ms, and the next spiking neuron was chosen at random. The firing rate was the same for all time bins (20 Hz). Likewise, the synaptic evolution encounters two phases. Alternating input spikes between *N*_1_ and *N*_2_ enhance the activity of *N*_3_ in the first place; therefore, both weight values initially slightly increase. When one presynaptic neuron takes the lead at random, the corresponding pre and postsynaptic spiking pattern establishes a strong causal correlation outweighing the other synapse, consequently reinforcing the chosen synapse. As a result, the highly probable anti-causal spike pairing for the unchosen synapse weakens the synapse. Similar to the case shown in Fig. 5b, two types of trajectories (Fig. 6b) were observed at random with equal probabilities.

We finally justified the feasible use of the proposed synaptic circuit for supervised learning. To this end, an additional presynaptic neuron (*N*_sup_) was deployed, which maintained a synaptic weight (*V*_w_sup_) of 50 mV and spiked at 50 Hz in sync with one of *N*_1_ and *N*_2_ (Fig. 6c and e). *N*_sup_ is termed as bias. Similar to the previous simulation, the activity (20 Hz) randomly toggled between *N*_1_ and *N*_2_ every 200 ms. A schematic of presynaptic spiking patterns in conjunction with a spiking pattern of *N*_sup_ in sync with *N*_1_ and *N*_2_ is depicted in Fig. 6c and e, respectively. This bias—as its name indicates—biases the vector field on the phase plane towards the side on which the weight of the out-of-sync neuron out of sync vanishes as plotted in Fig. 6d and f. Therefore, supervised learning can be achieved using the bias.

### Effect of MOSFET variability on STDP

MOSFET variability likely brings on a technical issue, particularly, for analog circuits. Bearing this in mind, we address the effect of such variability on STDP and the consequent selectivity evolution with regard to the robustness of the proposed synaptic circuit. MOSFET variability includes random dopant fluctuation (RDF) and line-edge roughness (LER)^46,47^. The former causes threshold voltage (*V*_th_) fluctuation that likely follows a normal distribution centered at the ideal *V*_th_ value (for invariant dopant density) with a standard deviation *σ*_*V*t_ given by the Pelgrom’s model^48^; ${\sigma }_{{V}_{{\rm{t}}}}={A}_{{\rm{RDF}}}/\sqrt{LW}$ where *A*_RDF_, *L*, and *W* denote a proportionality constant, channel length, and channel width, respectively. In this work, *A*_RDF_ was set to 1.27 × 10^−9^ V·m, conferring 15 mV in *σ*_*V*t_ on the smallest MOSFETs (60 nm × 120 nm) in line with ref.^47^. Additionally, LER was taken into account by allowing random variation in MOSFET channel length; the length for each MOSFET was drawn from a normal distribution with a standard deviation of ${A}_{{\rm{LER}}}/\sqrt{W}$ in which *A*_LER_ was set to 1.04 × 10^−12^ m^3/2^. This LER effect results in approximately 3 nm standard deviation for 120 nm channel in line with ref.^47^.

**Figure 7:**
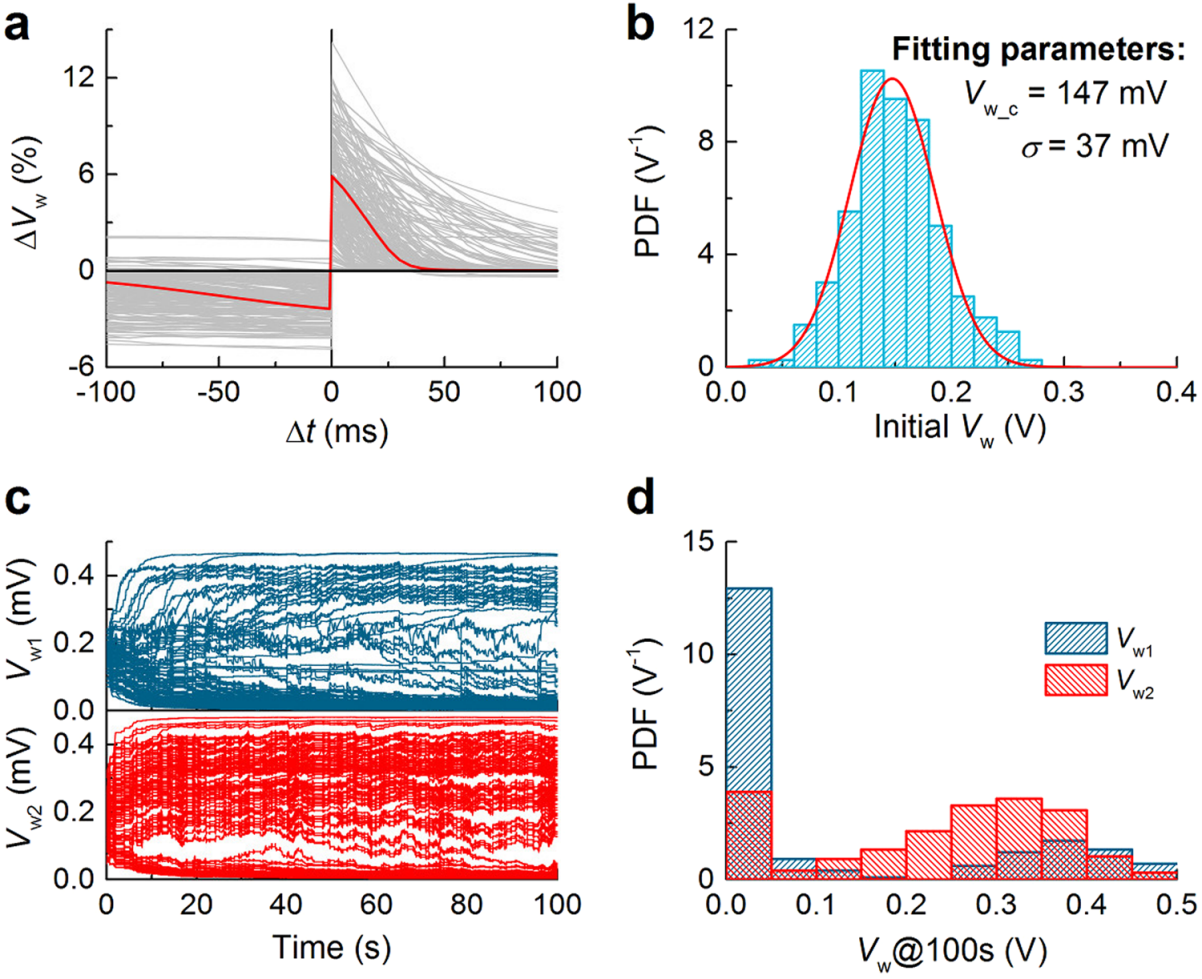
Effect of MOSFET variability including RDF and LER on STDP. (**a**) STDP behavior for 200 Monte Carlo simulation results (gray) and no variability (red). Each behavior was obtained with a different set of MOSFET parameters. (**b**) Distribution of initial synaptic weight, plotted from 200 Monte Carlo runs. (**c**) Synaptic evolution for 200 circuits given random MOSFET mismatch. The protocol in use was identical to that in Fig. 6(e) in attempt to lead *V*_w2_ to potentiation. (**d**) The distribution of *V*_w1_ and *V*_w2_ at 100 s.

Given these possible causes of variability, 200 pairs of synaptic circuits were acquired and subject to STDP and selectivity evolution identifications. In Fig. 7a, the 200 STDP behaviors (gray curves) are appended to the ideal one (red curve) identical to Fig. 3. Despite the present variability, the spike-timing effect (LTP and LTD for Δ*t* > 0 and Δ*t* < 0, respectively) is validated as a whole other than few exceptions. Alongside this spike-timing effect, the distribution of initial synaptic weight is of concern in selectivity evolution. The probability density function (PDF) of initial weight from 200 circuits is nicely fitted to a normal distribution function as plotted in Fig. 7b.

Eventually, selectivity evolution was identified for 200 pairs of circuits using the supervised learning scheme depicted in Fig. 6e—*N*_sup_ in sync with *N*_2_ to let *V*_w2_ win *V*_w1_. The data are plotted in Fig. 7c that reveals *V*_w2_ outweighing *V*_w1_ as a whole albeit scattered. Additionally, variation in initial synaptic weight can be ascertained in Fig. 7c. Figure 7d displays a PDF for *V*_w1_ and *V*_w2_ values at 100 s. It is noticeable that the potentiation probability of *V*_w2_ is higher than *V*_w1_ in support of the supervised learning.

## Discussion

**Figure 8:**
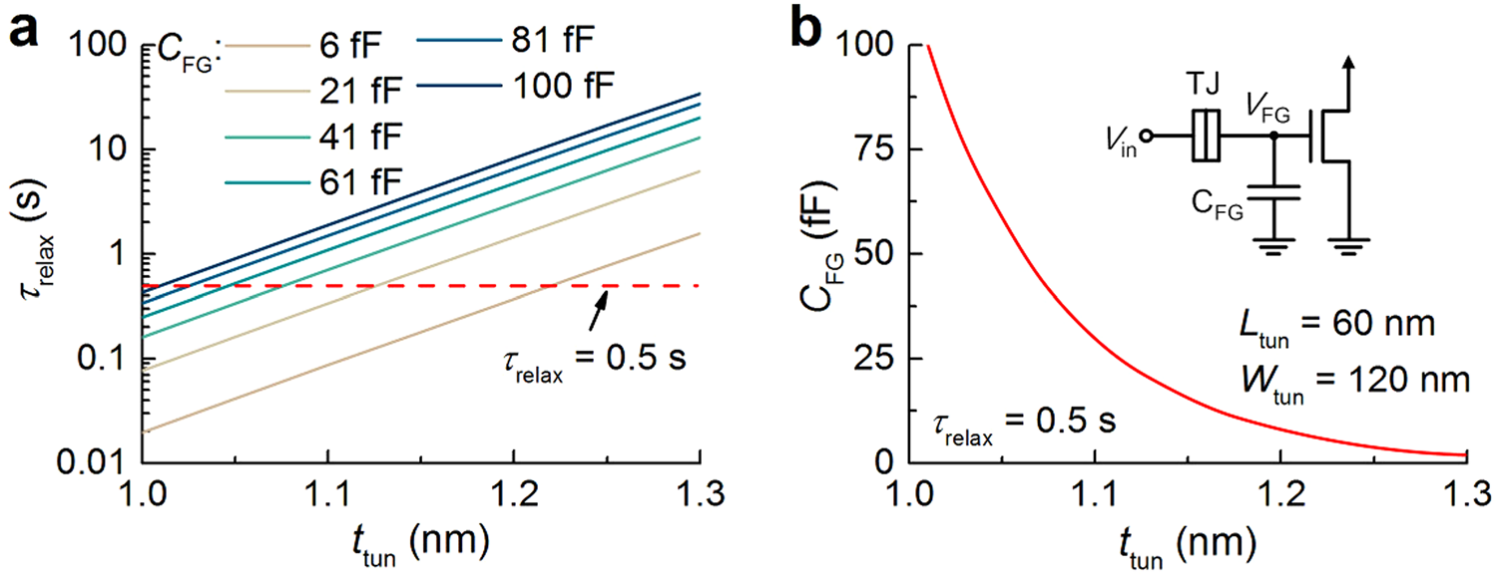
(**a**) Charge relaxation time *τ*_relax_ with tunnel barrier thickness *t*_tun_ for different capacitance *C*_FG_ values in the FG integrator sketched in the inset of (**b**). (**b**) A relationship between *C*_FG_ and *t*_tun_ for a *τ*_relax_ of 0.5 s.

The FG-based leaky integrators in the proposed synaptic circuit alleviate the area overhead for real-time scale operation in favor of scalability^10^. The FG-based leaky integrator may offer an advantage over the switched-capacitor integrator for deep submicron technology where the subthreshold leakage through the short channel^29–31^ is in need of high capacitance to enable real-time scale operation. To back the scalability of the FG-based integrator (e.g. TJ1 + C1 + M2 in Fig. 1) in part, its relaxation time was evaluated for different capacitances (*C*_FG_) and barrier thicknesses (*t*_tun_) (Fig. 8). Here the FG-based integrator was subject to a single spike (0.5 V amplitude and 30 μs width). The relaxation time *τ*_relax_ was defined as a time period during which the FG voltage amplitude falls below the half of its peak value. As such, *τ*_relax_ is remarkably susceptible to *t*_tun_ to the extent that almost two orders of magnitude change in *τ*_relax_ is managed by merely 30% change in *t*_tun_ while a rise in *C*_FG_ by approximately one order of magnitude increases *τ*_relax_ by less than two orders of magnitude (Fig. 8a). Figure 8b shows a *C*_FG_-*t*_tun_ relationship for a *τ*_relax_ of 0.5 s, indicating that 1.3 nm *t*_tun_ needs merely 2 fF *C*_FG_ for real-time scale operation.

**Figure 9:**
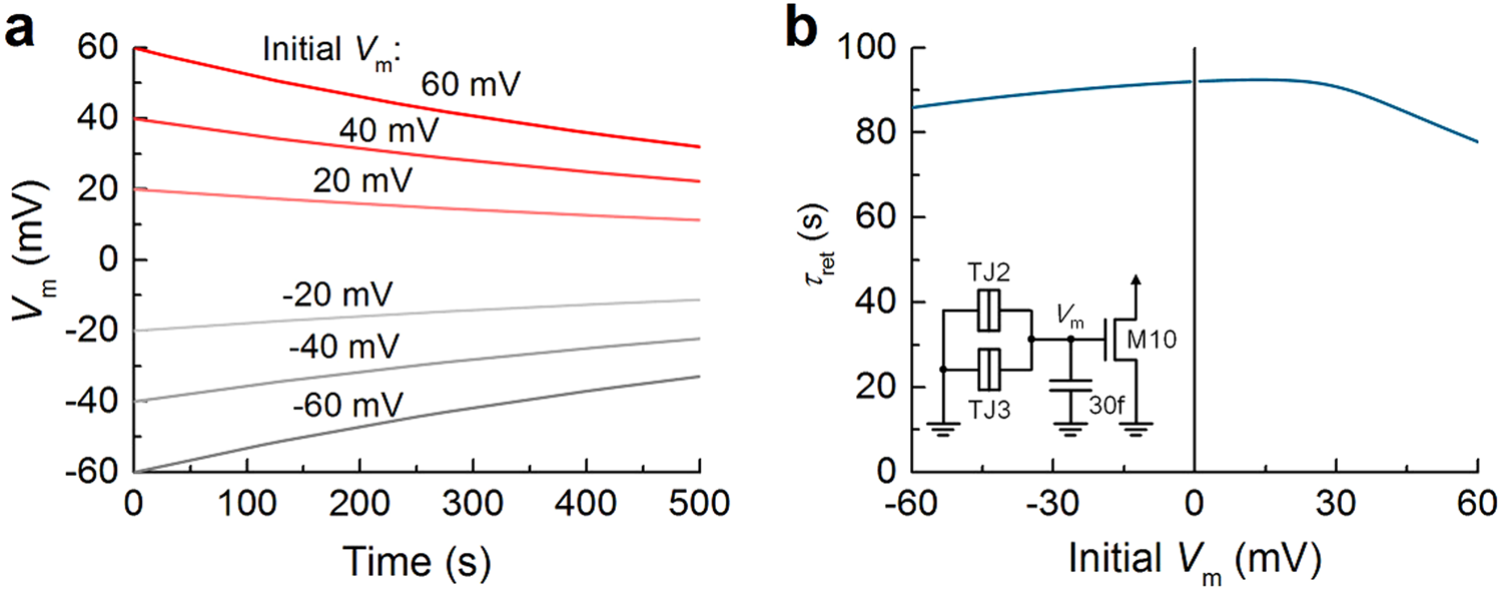
(**a**) Time-dependent change in *V*_m_ that was initially set to different values. (**b**) Retention time *τ*_ret_ for different initial *V*_m_ values. The retention time was defined as the time period during which |*V*_m_| decreases by 10%. The storage element in Fig. 1 was re-sketched in the inset.

The same is applied to the synaptic weight storage (TJ2 + TJ3 + C2 + M10), the expected relaxation time is much longer though. Hereafter, it appears proper to term this relaxation time as retention time. A thicker tunnel barrier is desirable in favor of a better retention; however, as such, charge injection through a thick tunnel barrier is of difficulty with regard to the tunneling probability that decays exponentially with barrier thickness. To be precise, it turns out that the spike (0.5 V amplitude and 30 μs width)—that employed through the entire simulations—cannot drive tunneling through a tunnel barrier (>1.8 nm). Thus, we chose 1.75 nm, which offers the retention of *V*_m_ programmed at different levels as shown in Fig. 9a. The retention time *τ*_ret_ was defined as a time period during which |*V*_m_| declines by 10%. *τ*_ret_ depends upon the programmed *V*_m_ level, which is typically in the 70–100 s range (Fig. 9b). The retention is perhaps insufficient for long-term memory. However, it appears feasible for synaptic competition (bifurcation) to terminate within the retention time as identified in Fig. 5a. In addition, if the memory retention is of significance for a particular application, it can be stored as binary numbers^25,26,32^.

The reliability of FG-MOSFETs is an important issue since the tunnel barrier is typically so thin that the programming voltage causes a high electric field across the barrier—that often brings on dielectric breakdown. Dielectric breakdown is often parameterized by charge-to-breakdown (QTB)^49–51^. QTB increases with decreasing the operating voltage; for silicon oxide layers (<3 nm) at 2.5 V, it was shown to exceed 10^6^ C/cm^2^ under constant voltage stress^52–54^. In our simulations, a single spike (0.5 V) to TJ1 and TJ4 drives <46 μC/cm^2^ and <4 μC/cm^2^, respectively. For TJ2 and TJ3, the value is below 1 μC/cm^2^. Therefore, the operation conditions partly support high endurance.

The observed variation in synaptic evolution due to MOSFET variability seemingly falls short of being accepted in a deterministic system without error-tolerance. However, benchmarking deep learning^55^, even a deterministic learning algorithm, such as back-propagation, needs to involve stochasticity in the beginning (initially random weight values) and during the training (regularization) for a better training^56^. Likewise, the stochasticity shown in the STDP behavior likely provides the network with high entropy (Shannon information) that allows a large number of representations. In line with deep neural networks, spiking neural networks may be in general error-tolerant such that the stochastic STDP shown in Fig. 7d may be acceptable to the extent to which the stochasticity does not lead to faulty results. Nevertheless, the degree of error-tolerance varies upon the architecture, learning rule, neuron model, etc., which is beyond the scope of this work. Thus, we leave this question open for the time being.

Energy efficiency is an important principle of neuromorphic engineering. The proposed synaptic circuit is energy-efficient with regard to the subthreshold operation of most MOSFETs in the circuit. The circuit theoretically consumes approximately 34 pW, and this power is almost identical to the standby power. Namely, the standby power consumption is dominant over the synaptic operational power consumption. Akin to the STDP behavior, the power consumption is also susceptible to MOSFET variability; the PDF of power consumption follows a normal distribution centered at 34 pW with a standard deviation of approximately 12 pW. This is fairly comparable to previous reports, for instance, 60 pW in the work by Bamford *et al*.^17^ and 37 pW by Cruz-Albrech *et al*.^28^.

**Figure 10:**
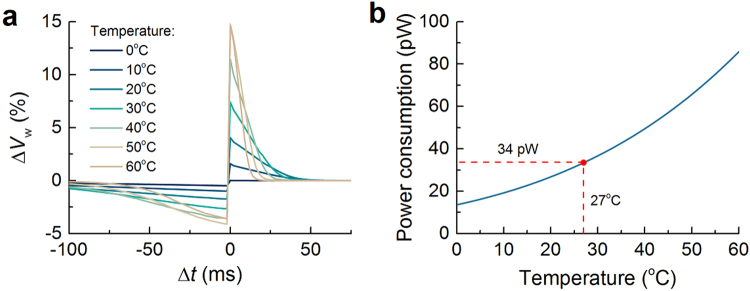
(**a**) STDP behavior at different circuit temperatures (0–60 °C). (**b**) Change in power consumption upon the circuit temperature.

The temperature-resilience of the proposed synaptic circuit should also be taken into consideration. Practically, the circuit varies on its temperature (mostly, temperature increase) due mainly to power dissipation on the chip and/or ambient temperature change. To identify the temperature resilience, we varied the circuit temperature from 0 to 60 °C and acquired the STDP behavior at each temperature. The simulation results reveal that the STDP behavior is as a whole preserved in the given temperature range in spite of the variation in detail (Fig. 10a). The detail is addressed in Supplementary Information. Further, the power consumption increases with circuit temperature, reaching approximately 86 pW at 60 °C as shown in Fig. 10b. The increase is due mainly to the subthreshold operation of all MOSFETs in the circuit in that the channel current is thermally activated, and thereby consuming more power.

Given the significantly low power operation of the synaptic circuit, the power consumption and consequent temperature increase are unlikely to be sufficiently high to heat the circuit above 60 °C even on the synaptic array level. As a benchmark, the recent central processing unit (Intel i7-6700K) under the maximum load consumes approximately 100 W and its core reaches approximately 70 °C when cooled by air. Thus, we believe that the temperature of the synaptic array with air-cooling stays much below this benchmark given the extremely low power consumption of a single synaptic unit.

The estimated circuit area is approximately 40 µm^2^, whose layout is shown in Fig. S4 in Supplementary Information. When implemented in a crossbar array, the area is reduced down to 24 µm^2^ such that the unit synaptic circuits in the same row and column can share the capacitors. Other synaptic circuit designs are nicely overviewed in ref.^20^. Notably, the three capacitors take a considerable portion (ca. 35%) so that the use of a high-*k* dielectric material in place of SiO_2_ is a solution to a reduction in the circuit area.

## Conclusion

We proposed a synaptic circuit for STDP, which potentially fulfills competitive synaptic adaptation (selectivity) with randomly spiking neurons at significantly low expense (area overhead and power consumption). This outstanding potential of the proposed circuit mainly owes to the FG integrator for the state variables and synaptic weight storage, which is expected to outperform capacitor-based integrator, particularly, in the deep-submicron regime. In this study, the STDP was viewed as the reinforcement of causality of postsynaptic spiking. In this regard, the circuit simulation highlighted the spontaneous evolution of synaptic weight with regard to causality reinforcement in a random (unsupervised learning) and deliberate manner (supervised learning with the aid of bias).

## Electronic supplementary material

Supplementary Information
