# Supplementary material for: Scalable excitatory synaptic circuit design using floating gate based leaky integrators
Source: Sci Rep. 2017 Dec 14;7:17579. doi: 10.1038/s41598-017-17889-8 (PMC5730552; doi:10.1038/s41598-017-17889-8)
Supplement: Supplementary file 1 — Supplementary Information [file 41598_2017_17889_MOESM1_ESM.pdf]

Supplementary Information for

Scalable excitatory synaptic circuit design using floating gate based leaky integrators

Vladimir Kornijcuk,[1,2] Hyungkwang Lim,[1] Inho Kim,[1] Jong-Keuk Park,[1] Wook-Seong Lee,[1] Jung-Hae Choi,[1] Byung Joon Choi,[3] and Doo Seok Jeong*[1,2]

[1]Center for Electronic Materials, Korea Institute of Science and Technology, Seoul 02792, Republic of Korea

[2]Department of Nanomaterials, University of Science and Technology, Daejeon 34113, Republic of Korea

[3]Department of Materials Science and Engineering, Seoul National University of Science and Technology, Seoul 01811, Republic of Korea

*Correspondence and requests for materials should be addressed to D.S.J. (dsjeong@kist.re.kr)

SI1. Subcircuit-wise synaptic circuit operation

In the present circuit simulation, the tunnel junction was simulated by a MOSFET having source, drain, and body terminals short-circuited. Spikes are applied to the short-circuited terminal such that the charge is injected into the FG through the tunnel barrier (gate oxide in the model). Quantum mechanical electron tunneling through the tunnel barrier is provided by the BSIM 4.6.0 model that evaluates the tunneling current within the framework of the Wentzel-Kramers-Brillouin approximation[1,2,3]. The tunneling equation is semi-empirical with regard to the use of an auxiliary function that improves the accuracy of the original Fowler-Nordheim tunneling equation[4].

Electrostatic potential evolving upon charge transfer into and out of the FG self-consistently offers negative feedback on the transfer, providing the detailed balance. The detailed balance is the ground of the charge relaxation (essential to leaky integration) and limited growth of synaptic weight. To be precise, the positive charge piled up on the FG by a positive voltage to the TJ raises the FG voltage (V_{FG_post}, V_{FG_pre}, and V_m for M2, M11, and M10, respectively), and thus reduces the driving potential in due course under voltage application, inhibiting positive charge injection into the FG. That is, confining charge on the FG spontaneously lets the

depolarization field evolve against the applied polarization field. The same is applied to the case of relaxation (no voltage to the TJ); in this case the depolarization field solely drives charge transfer out of the FG. The same holds for the case of negative charge injection and ejection (TJ3+M10). The charge ejection is again governed by the ejection kinetics that can be controlled by engineered tunnel barrier with regard to the barrier height and thickness. Detailed charge relaxation behavior with different barrier thicknesses can be seen in a recently proposed leaky integrate-and-fire (LIF) neuron model based on a FG-integrator (reference 10 in the main text).

In contrast, sluggish charge relaxation (memory retention) is desirable for the storage such that the relaxation is retarded, mainly, using a thicker tunnel barrier. Given the interaction between wave functions in initial and final states, which exponentially decays with the barrier thickness, the charge relaxation kinetics through tunneling is susceptible to the barrier thickness. However, the same holds for charge injection kinetics such that the use of a thicker tunnel barrier raises the programming voltage; therefore, memory retention and moderate programming voltage need to be reconciled in the FG-MOSFET design. The proposed synaptic circuit uses operation voltage in the range -0.8 V – 0.5 V. In this regard, a barrier thickness of 1.75 nm appears to be optimal for 65 nm CMOS technology as used for TJ2 and TJ3 in Fig. 1.

The leaky integrator for postsynaptic state variable generation consists of two inverting common source stages (M1-M2 and M3-M4 in Figs. 1a and S1a). Unlike the first stage (conventional common source amplifier), two n-channel MOSFETs (nMOSFETs for short) form the second stage, enabling a rather gradual change in $V_{\mathrm{s\_post}}$ with $V_{\mathrm{FG\_post}}$ in its VTC as shown in Figs. S1b and c. As shown, the constant voltage V_{d1} and V_{d2} are in control of the VTC that is consequently related to the eventual STDP behavior. The sampling subcircuits in Figs. 1c and d sample the state variable upon an incident opposite spike. For sampling the postsynaptic state variable, a two-stage amplifier (M5-M8 in Fig. 1c) is used, which outputs voltage ($V_{\mathrm{bias\_post}}$) with input (V_{pre}) as shown in Fig. S1d. The output pulls up the drain of M3 to $V_{\mathrm{dd+}}$ (0.5 V); a non-zero state variable ($V_{\mathrm{s\_post}}$) is elicited given $V_{\mathrm{FG\_post}}$.

In contrast, the leaky integrator for postsynaptic state variable generation is a single inverting stage (M11-M12 in Figs. 1b and S1e). The additional header pMOSFET (M13) is for sampling the decaying state variable with the aid of the read-out subcircuit in Fig. 1d. As for V_{d1} and V_{d2} in the postsynaptic state variable generator, the VTC for the presynaptic state variable can be tweaked by V_{p1} and V_{p2} (Figs. S1f and g).

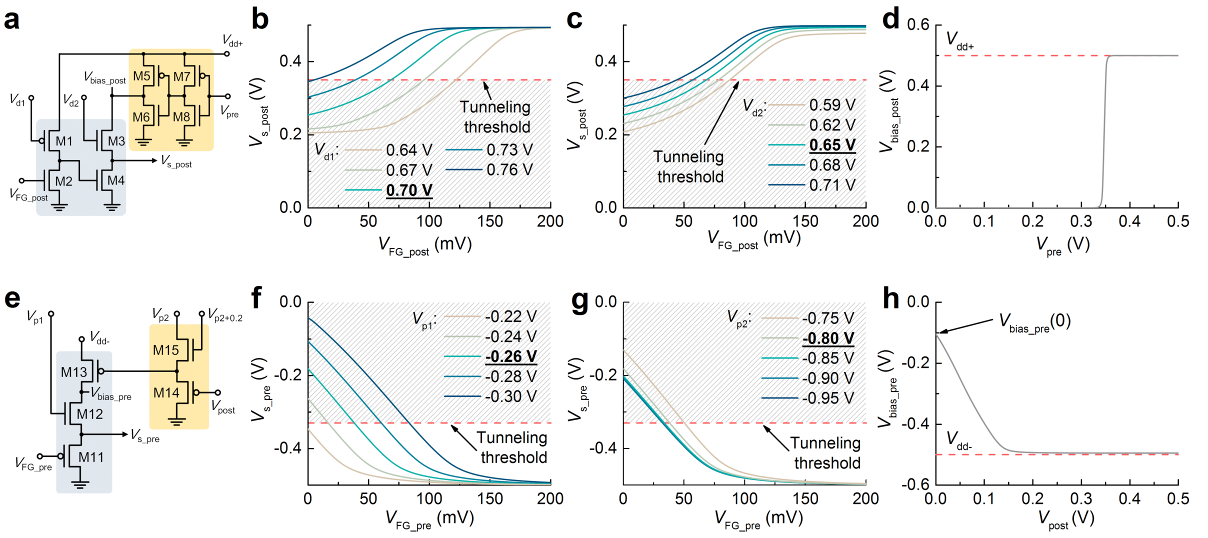

Figure S1. VTC of each state variable generator. (a) The postsynaptic state variable generator alongside the sampling subcircuit. VTC with V_{FG_post} at different (b) V_{d1} and (c) V_{d2}. V_{bias_post} was set to 0.5 V. (d) The VTC of the sampling subcircuit. (e) – (h) The same order of figures as (a) – (d) but for the circuit for the presynaptic state variable. Other than the control parameters, the parameters in Tables 2 and 3 were used for these simulations.

V_{s_post} and V_{s_pre} that capture the post and presynaptic state variable, respectively, are relayed to the storage M9-M10 through tunnel junctions TJ2 and TJ3, respectively. The contribution of the state variables to the overall synaptic weight change (ΔV_m) needs to be competitive such that V_{s_pre} is designed to be negative while V_{s_post} positive. V_m (FG voltage) accordingly evolves and outputs V_w that subsequently charges the membrane of the postsynaptic neuron. The larger V_w, the larger excitatory postsynaptic potential (EPSP) is caused, equivalent to synaptic weight. This storage circuit employs an additional control signal V_{ctrl} to shift the VTC (Fig. S3a), and thus to determine the initial weight (V_{w0}) as shown in Fig. S3b.

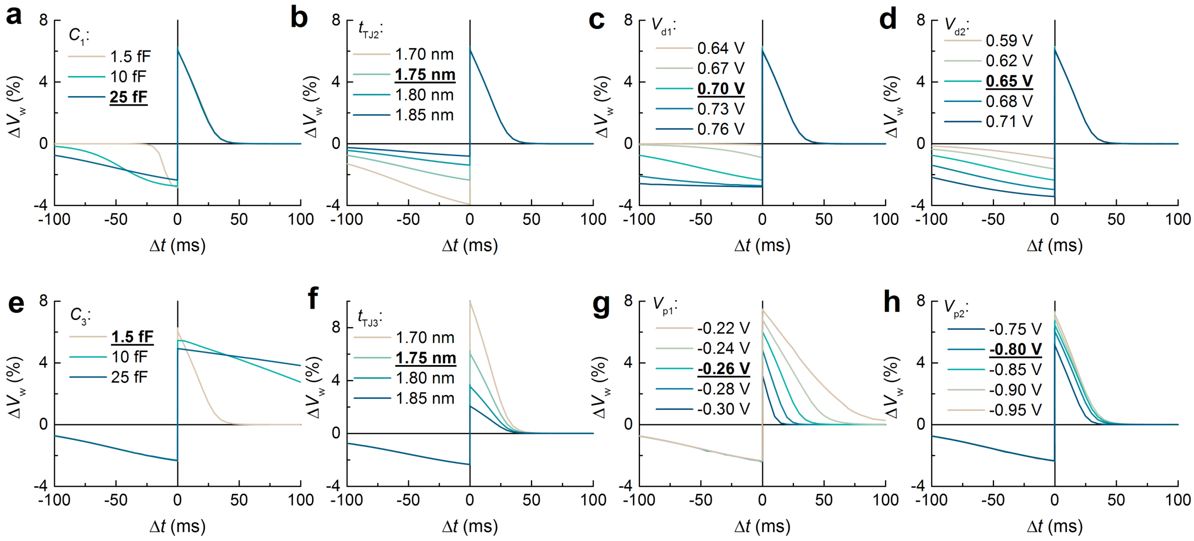

Figure S2. Tunability of STDP behavior by means of circuit parameters. The LTD behavior can be tweaked by parameters such as (a) C_1 and (b) t_{TJ2}, (c) V_{d1}, and (d) V_{d2}. Also, the LTP can be tweaked by parameters such as (e) C_3 and (f) t_{TJ3}, (g) V_{p1}, and (h) V_{p2}. The reference (initial) synaptic weight (V_{w0}) was 151 mV that results from a V_{ctrl} of 0.51 V.

SI2. Change in STDP behavior upon circuit parameters

The proposed synaptic circuit is versatile with regard to the tunability of the resulting STDP behavior by means of several parameters. It highlights needs for such tunability that biological STDP behaviors differ for different areas in the brain[5,6]. To this end, the proposed synaptic circuit provides several means of STDP tunability. First, the STDP behavior is largely reliant upon several physical parameters that offer the ground of circuit design, e.g. capacitance for leaky integrator (C_1, and C_3) and tunnel barrier thickness (t_{TJ1} and t_{TJ4}). Besides, several control signals, e.g. V_{d1}, V_{d2}, V_{p3}, and V_{p4}, enable post-fabrication tuning that renders the given circuit reconfigurable. The effect of each parameter on the resulting STDP behavior is plotted in Fig. S2.

Moreover, the weight-depending ΔV_w can readily be adjusted by means of the control voltage V_{ctrl} that shifts the VTC of M9+M10 (Fig. S3a). The VTC reveals that the higher V_{ctrl} is applied, the lower V_m sufficiently outputs the same V_w, and thus the FG needs to be loaded with less positive charge. Therefore, $|\Delta V_w|$ by V_{s_pre} ($\Delta V_w < 0$) for the same single pre-postsynaptic spike pair tends to decline with V_{ctrl}. In contrast, $|\Delta V_w|$ by V_{s_post} ($\Delta V_w > 0$) tends to increase with V_{ctrl} in view of the detailed balance of charge transfer. In this regard, the weight-

dependence of STDP behavior markedly depends upon V_{ctrl}. Also, V_{ctrl} alters the initial synaptic weight (V_{w0}). Therefore, the synaptic bifurcation kinetics remarkably differs for the different V_{ctrl} values as shown in Fig. S3d, particularly, the saturation weight values, which leaves this means very effective in adjusting synaptic behavior with regard to synaptic selectivity evolution.

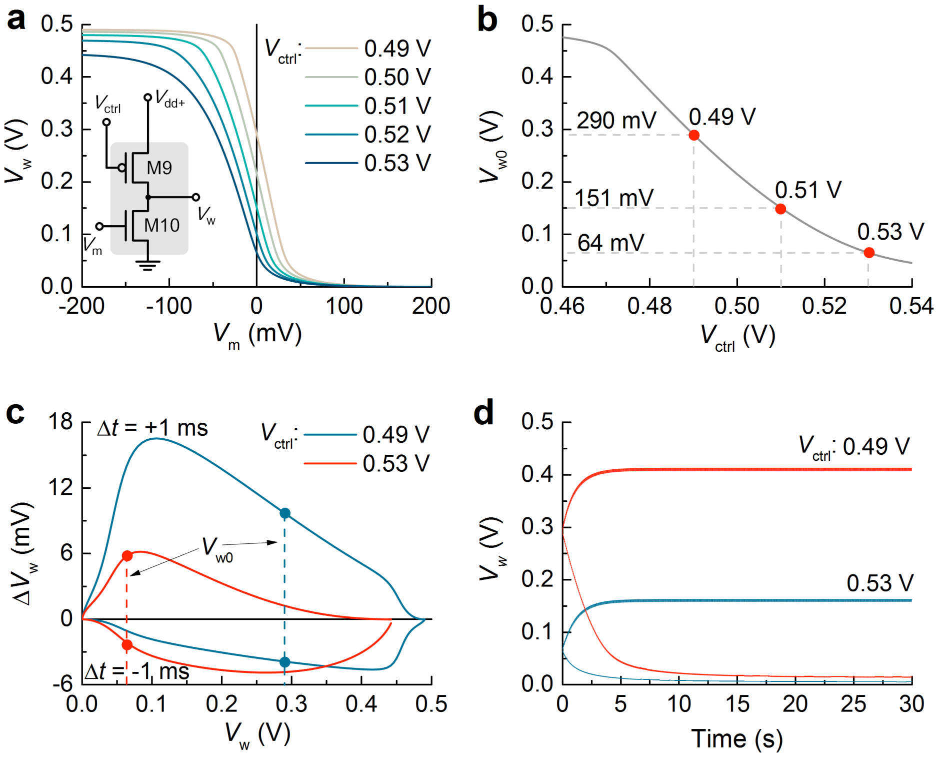

Figure S3. (a) VTC of the storage subcircuit (inset) at different control voltage V_{ctrl} in the 0.49 – 0.53 V range. (b) Initial synaptic weight V_{w0} with respect to V_{ctrl}. (c) Dependence of ΔV_w on V_w when the synaptic circuit is subject to a single pair of pre and postsynaptic spikes with Δt = ±1 ms at two different V_{ctrl}. (d) Consequent synaptic bifurcation.

SI4. Performance dependence on operating temperature

The simulated temperature effects notably include (i) the increase in ΔV_w upon each update and (ii) decrease in both LTP and LTD timing windows with temperature. The first effect arises in part from the temperature-induced change in VTC for the subcircuits in Figs. 1a and b (Figs. S4a and b). The change in VTC is attributed to the subthreshold operation of all MOSFETs in the circuit in that the channel conductance remarkably depends on the circuit temperature (Fig. S4c). The temperature effect on VTC ascertains the increase in above-threshold V_{s_post} and V_{s_pre} at a given V_{FG_post} and V_{FG_pre}, respectively, with temperature, which

consequently results in ΔV_w with temperature. The kinetics of voltage change also accounts for the temperature dependence of ΔV_w (Figs. S4e and f). The transient V_{s_pre} markedly varies upon temperature due mainly to the pMOSFET voltage divider (M13). This voltage divider is sufficiently resistive for noticeable RC delay in the given time domain, and thus a reduction in the delay with temperature is obvious. The second effect is attributed to the thermal activation of charge ejection out of the floating gate and consequent decrease in FG voltage relaxation time (Fig. S5). Such a decrease in relaxation time narrows down the STDP timing windows. Nevertheless, the temperature effect barely deprives the circuit of STDP behavior, supporting the temperature-resilience of the proposed circuit.

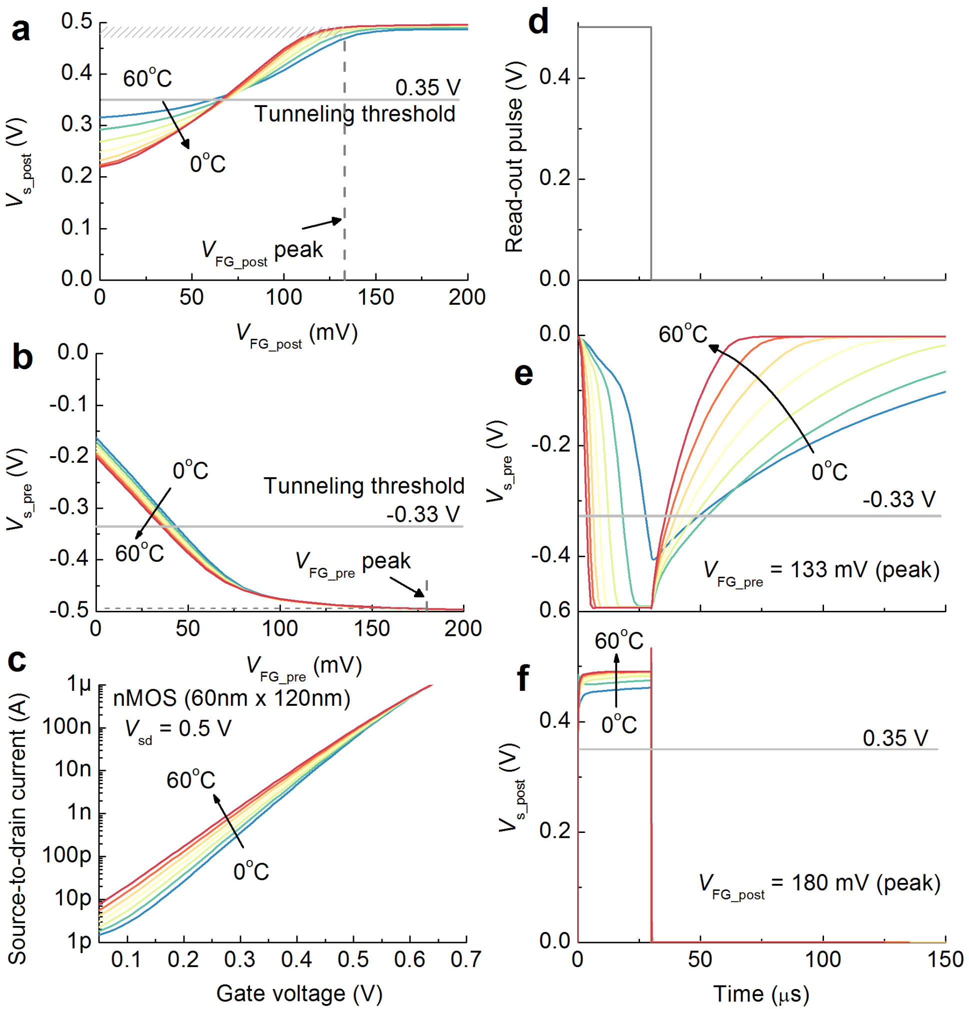

Figure S4. Temperature-varying VTC for the subcircuits in (a) Figs. 1a and (b) 1b. V_{FG_post} and V_{FG_pre} peaks denote the maximum V_{FG_post} and V_{FG_pre} reached upon incident post and presynaptic spikes, respectively. (c) Temperature effect on source-to-drain current of a subthreshold nMOS. The time-dependent (e) V_{s_pre} and (f) V_{s_post} behaviors in response to V_{pre} and V_{post} pulses (i.e. spikes) shown in (d).

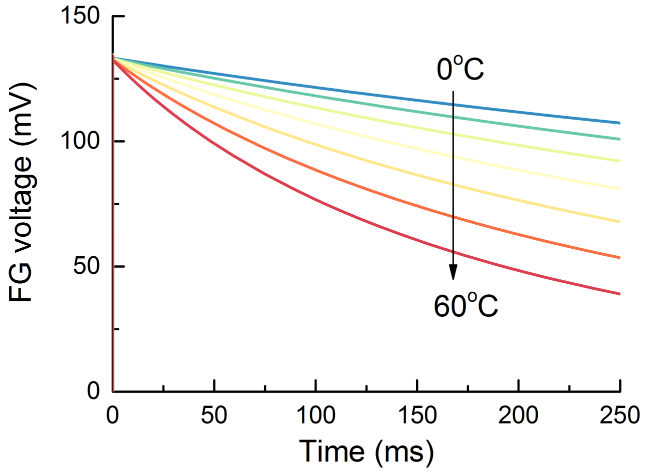

Figure S5. Due course decay of FG voltage at different temperatures.

SI5. Circuit layout

A layout of the proposed synaptic circuit is shown in Fig. S6. It was designed using Electric[7]. The sizes of MOSFETs in Table 3 were used. For the capacitors, a capacitance density of 4.1 fF/μm^2 was considered, which is for dual MIM capacitors that are provided by foundries.

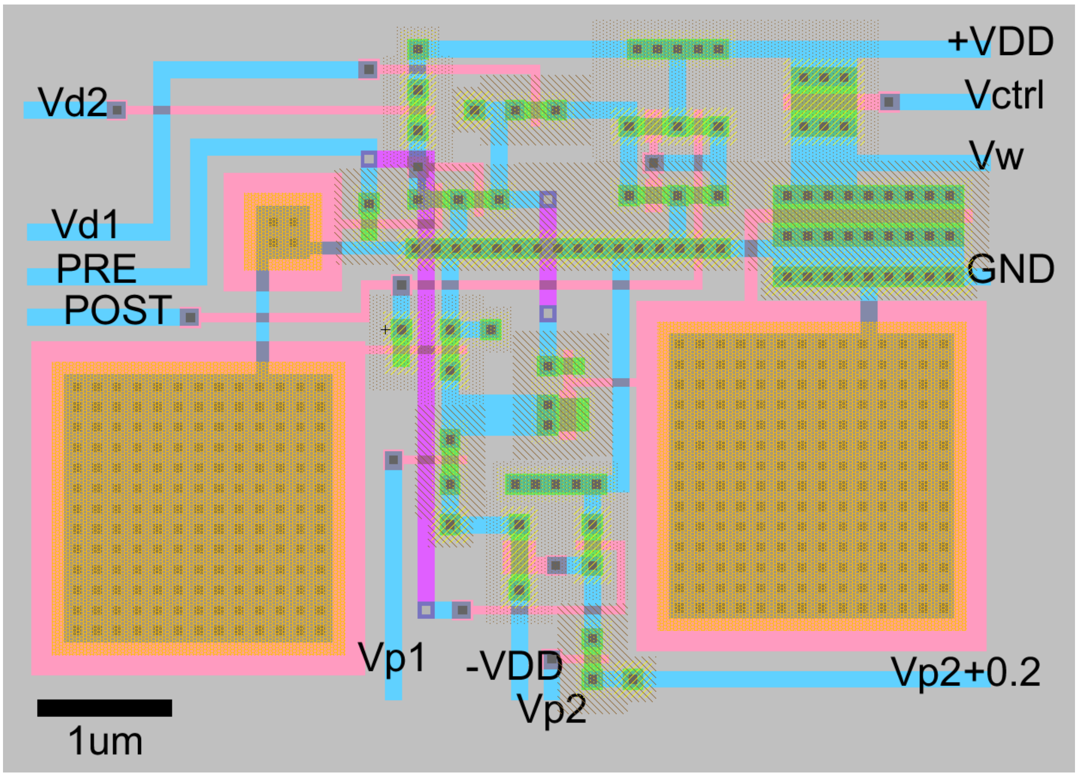

Figure S6. Layout of the proposed synaptic circuit.

1. Dunga, M. et al. *BSIM4.6.0 MOSFET Model*. University of California (2006).

2. Cao, K. et al. BSIM4 gate leakage model including source drain partition. *Tech. Dig. Int. Electron Devices Meeting*, 815-818 (2000).

3. Lee, W. & Hu, C. Modeling CMOS tunneling currents through ultrathin gate oxide due to conduction- and valence-band electron and hole tunneling. *IEEE Trans. Electron Devices* 48, 1366-1373 (2001).

4. Ranuárez, J., Deen, M. & Chen, C. A review of gate tunneling current in MOS devices. *Microelectron. Reliab.* 46, 1939-1956 (2006).

5. Dan, Y. & Poo, M. Spike timing-dependent plasticity: From synapses to perception. *Physiol. Rev.* 86, 1033-1048 (2006).

6. Sjöström, P. & Häusser, M. A cooperative switch determines the sign of synaptic plasticity in distal dendrites of neocortical pyramidal neurons. *Neuron* 51, 227-238 (2006).

7. Rubin, S. M, An integrated aid for top-down electrical design. Proc. VLSI83, 63-72 (1983).
